# Supplementary figures and images for: RadD from Fusobacterium nucleatum engages NKp46 to promote antitumor cytotoxicity
Source: eLife. 2026 May 1;14:RP108439. doi: 10.7554/eLife.108439 (PMC13134850; doi:10.7554/eLife.108439)

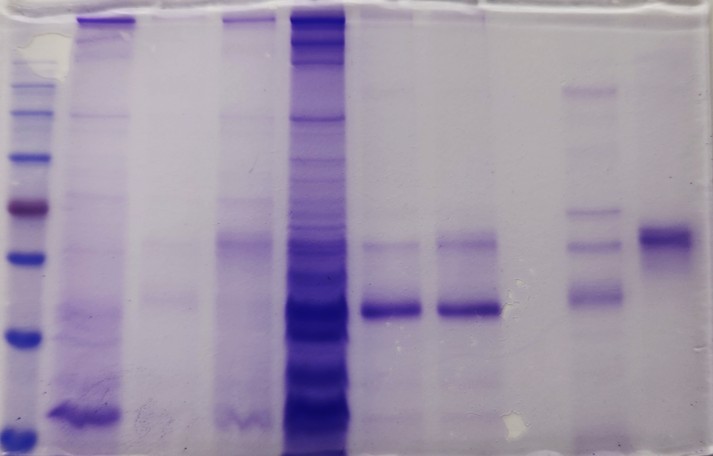

Supplement: Figure 2—source data 1. [file elife-108439-fig2-data1.zip › Figure 2 Source data 1/Figure 2 Source data 1 Uncropped and unlabeled gel.jpg]
