# Supplementary material for: RadD from Fusobacterium nucleatum engages NKp46 to promote antitumor cytotoxicity
Source: eLife. 2026 May 1;14:RP108439. doi: 10.7554/eLife.108439 (PMC13134850; doi:10.7554/eLife.108439)
Supplement: Figure 2—source data 2. [file elife-108439-fig2-data2.zip › Figure 2 Source data 1 - labeled/Figure 2 Source data 1 labeled.pdf]

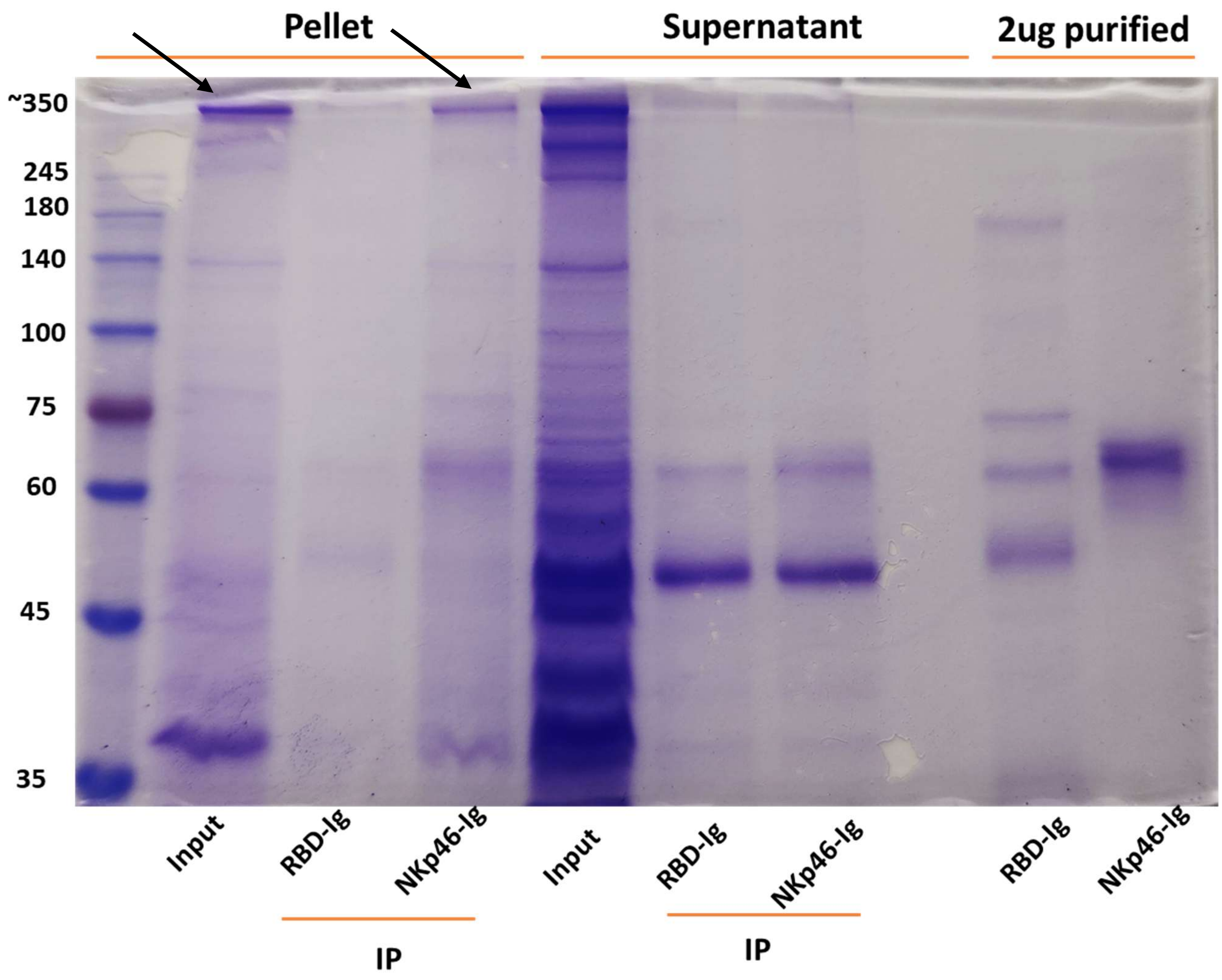

Figure 2, Source Data 1. Original Western blot validating the interaction between Fusobacterium nucleatum and NKp46. An immunoprecipitation assay was performed using an NKp46–Ig fusion protein with F. nucleatum subsp. nucleatum ATCC 23726. Molecular weight markers are shown on the left. Lane 1 (Input) contains total lysates from the bacterial pellet (membrane protein fraction), showing a band corresponding to RadD (~350 kDa)-arrow. Lane 2 (RBD–Ig control) shows immunoprecipitation with 2.5 µg of control RBD–Ig, with no detectable band at ~350 kDa. Lane 3 (NKp46–Ig) shows immunoprecipitation with 2.5 µg of NKp46–Ig, revealing a band at ~350 kDa-arrow. Lanes 4–6 correspond to the supernatant fraction of the bacterial lysate. No bands are observed in lanes 5 and 6, indicating a lack of interaction in this fraction. Lanes 7 and 8 contain 2.5 µg of purified RBD–Ig and NKp46–Ig proteins, respectively.
